# Geographic consistency in dominant, non-typeable *Haemophilus influenzae* genotypes colonising four distinct Australian paediatric groups: a cohort study

**DOI:** 10.1186/s41479-016-0013-y

**Published:** 2016-08-18

**Authors:** Heidi C. Smith-Vaughan, Jemima Beissbarth, Jacinta Bowman, Kim M. Hare, Erin P. Price, Janessa Pickering, Deborah Lehmann, Anne B. Chang, Peter S. Morris, Robyn L. Marsh, Amanda J. Leach

**Affiliations:** 1 Menzies School of Health Research, Charles Darwin University, Darwin, Northern Territory Australia; 2 School of Medicine, Griffith University, Gold Coast, Queensland Australia; 3 Department of Microbiology and Infectious Diseases, PathWest Laboratory Medicine, Perth, Western Australia Australia; 4 Telethon Kids Institute, The University of Western Australia, Perth, Western Australia Australia; 5 School of Paediatrics and Child Health, University of Western Australia, Perth, Western Australia Australia; 6 Department of Respiratory and Sleep Medicine, Queensland Children’s Medical Research Institute, Children’s Health, Queensland University of Technology, Brisbane, Queensland Australia; 7 Royal Darwin Hospital, Darwin, Northern Territory Australia

**Keywords:** Non-typeable *Haemophilus influenzae*, Genotyping, Carriage, Paediatric, PCR-ribotyping

## Abstract

Non-typeable *Haemophilus influenzae* (NTHi)-associated ear and respiratory diseases (including pneumonia) represent a major health burden in many parts of the world. NTHi strains retrieved from the upper airways commonly reflect those found in the lower airways. Despite growing genomic and genotyping data on NTHi, there remains a limited understanding of global and regional NTHi population structures. The aim of this study was to determine whether nasopharyngeal carriage in four Australian paediatric groups at varying risk of NTHi colonisation was dominated by the same NTHi genotypes. Genotyping data generated by PCR-ribotyping were evaluated for 3070 NTHi isolates colonising the nasopharynges of Aboriginal and non-Aboriginal children enrolled in four longitudinal studies in three separate urban and remote regions of Australia. Several NTHi PCR-ribotypes dominated in nasopharyngeal carriage, irrespective of study setting. Principal coordinates analysis confirmed a cluster of common PCR-ribotypes among all cohorts. In conclusion, we identified dominant PCR-ribotypes common to geographically disparate Australian paediatric populations. Future genomic analyses will shed further light on the precise factors underlying the dominance of certain NTHi strains in nasopharyngeal carriage.

## Background

Diseases associated with non-typeable *Haemophilus influenzae* (NTHi) represent a major health burden worldwide [1]. This Gram-negative bacterium is commonly associated with adult community-acquired pneumonia (CAP), particularly among those with underlying respiratory disease [2]. Because young children generally cannot expectorate, the aetiology of paediatric CAP is poorly understood and a role for NTHi remains controversial. NTHi is also associated with chronic lung diseases; for example, it is the most commonly detected pathogen in chronic obstructive pulmonary disease, a disease identified by the World Health Organization as the third leading cause of mortality globally [3]. NTHi is the most common bacterium in the lower airways of adults and children with non-cystic fibrosis bronchiectasis [4, 5], being cultured from the sputa of up to 70 % of adults [5], and 78 % of bronchoalveolar lavage specimens from Australian Indigenous children with bronchiectasis [4]. NTHi is also a major otitis media pathogen and has been detected in 89 % of ear discharge specimens from children with suppurating ears in remote Australian Indigenous communities [6].

Genetic and genomic studies have shown that NTHi is a genetically diverse organism that frequently undergoes lateral gene transfer [7, 8], a trait that has hindered our understanding of the population biology of this organism. As an example of this diversity, the *H. influenzae* multilocus sequence typing (MLST) database (http://pubmlst.org/hinfluenzae/) currently lists 1369 NTHi isolates with MLST data from across the globe, which are represented by 966 distinct sequence types (STs). Similarly, our studies of NTHi nasopharyngeal carriage isolates have identified 130 PCR-ribotypes (PRTs) to date. Despite this high level of diversity, several studies have identified a population structure among NTHi [7, 9]. Of note, MLST and whole genome analyses show some correlation between population structure and virulence potential, although no geographic clustering was detected [7, 9]. While these data contribute to our understanding of NTHi populations, large genomic studies are necessary to improve our understanding of geographic patterns, temporal changes (including outbreaks), adaptive evolutionary pressures such as those driven by antibiotic or vaccine selection, and tissue tropism. Whole genome sequencing is ideal for exploration of bacterial population structure, and that is the direction for future studies; however, in the current study we used PRT. In addition to its cost effectiveness, PRT has the advantage of interrogating 16S rDNA, which is an evolutionarily stable marker across bacterial species. This slow rate of evolution potentially provides a more robust signal for detecting strain relatedness than other genotyping methods that are based on non-ribosomal loci. We compared PRT data for 3070 nasopharyngeal carriage NTHi isolates obtained from four paediatric groups from three distinct geographic regions of Australia. Our aim was to determine whether the same NTHi PRTs dominated in nasopharyngeal carriage in these geographically distinct populations.

## Methods

### Study design

This was a retrospective cohort study that analysed NTHi genotyping data generated previously in completed studies.

### Participants and setting

Study design, setting (location, dates of data collection, child eligibility criteria) are summarised in Table 1. Written informed consent was obtained from families to enrol their child in these studies. Consent forms and procedures were undertaken according to requirements of each ethical review board (see details below). Participants in Study 1 (Table 1) were primarily non-Aboriginal children attending childcare in the tropical city of Darwin in the Northern Territory; these children were enrolled in a cluster randomised-controlled trial of a hygiene intervention [10]. All families were invited by letter to participate and those children whose parents consented were included in the study. Study 2 participants were Aboriginal children with acute otitis media (AOM) living in 16 remote communities across the Northern Territory; these communities ranged from those in the arid centre of Australia, up to the tropical northern “Top End” region. We attempted to screen all age-eligible children for AOM. Children with AOM whose parents consented were enrolled in a randomised-controlled trial of antibiotics for AOM [11]. Study 3 and Study 4 included Aboriginal and non-Aboriginal children, respectively, from the semi-arid town of Kalgoorlie in southern Western Australia enrolled in a prospective study of otitis media and nasopharyngeal carriage [12]. All mothers were visited post-partum and invited to participate. None of the children in any of the four study groups (cohorts) had received the 10-valent pneumococcal *H. influenzae* protein D vaccine.

**Table 1:** Description of the 4 Australian paediatric non-typeable *Haemophilus influenzae* (NTHi)-carriage cohorts used in this study

Study	Study design, setting and participant ethnicity	Year of data	No. enrolled children (NTHi carriage rate)	Child eligibility age & other key criteria^b^	Collection frequency (No. NTHi positive swabs)	No. unique NTHi typed^a^ (Total NTHi typed)	No. PRTs (No. per 100 swabs)
1	Cluster RCT of a hygiene intervention in 20 Darwin (NT) child care centres. 90 % were non-Aboriginal [10]	2001	456 (50 %)	0–4 yrs, attending 3 d/wk	Fortnightly for 6 m (2,012)	2,179 (2,201)	84 (4)
2	RCT of azithromycin *vs* amoxicillin for AOM in 16 remote NT communities. All were Aboriginal children [11]	2003–2005	320 (316 with swab data) (85 %)	0.5–6 yrs with AOM	Days 0 and 6–11. Additionally, Day 12–21 if perforation. (469)	551 (569)	73 (13)
3	Kalgoorlie (WA) Otitis Media Research Project. Prospective Aboriginal cohort [12, 14]	1999–2005	100 (36.3 %)	1 wk to 24 m	Ages 1–3, 6–8 wks, then months 4, 6, 12, 18, 24. (193)	231 (346)	65 (28)
4	Kalgoorlie (WA) Otitis Media Research Project. Prospective non-Aboriginal cohort [12, 14]	1999–2005	180 (9.4 %)	1 wk to 24 m	Ages 1–3, 6–8 wks, then months 4, 6, 12, 18, 24. (102)	109 (192)	37 (34)

*RCT* randomised-controlled trial, *AOM* acute otitis media, *NP* nasopharyngeal, *PRT* PCR-ribotype, *NT* Northern Territory, *WA* Western Australia

^a^A single isolate of each PRT from each swab

^b^All families provided written informed consent for their child’s participation

### Laboratory and statistical analyses

For this study, we used genotyping data generated by PRT, a method that interrogates genetic polymorphisms in the *H. influenzae* ribosomal operons using restriction digestion [13]. Briefly, approximately 6 kb ribosomal operons (16S–5S) were amplified by long-PCR, and *Hae*III restriction fragments of the resulting product were separated by agarose gel electrophoresis. PRTs were assigned based on restriction fragment lengths (0.4 to 1.2 kb) following alignment to a 2-log DNA ladder (New England BioLabs, United States) and an in-house standard. To aid pattern recognition, the images were divided into 16 predetermined sections.

**Fig. 1:**
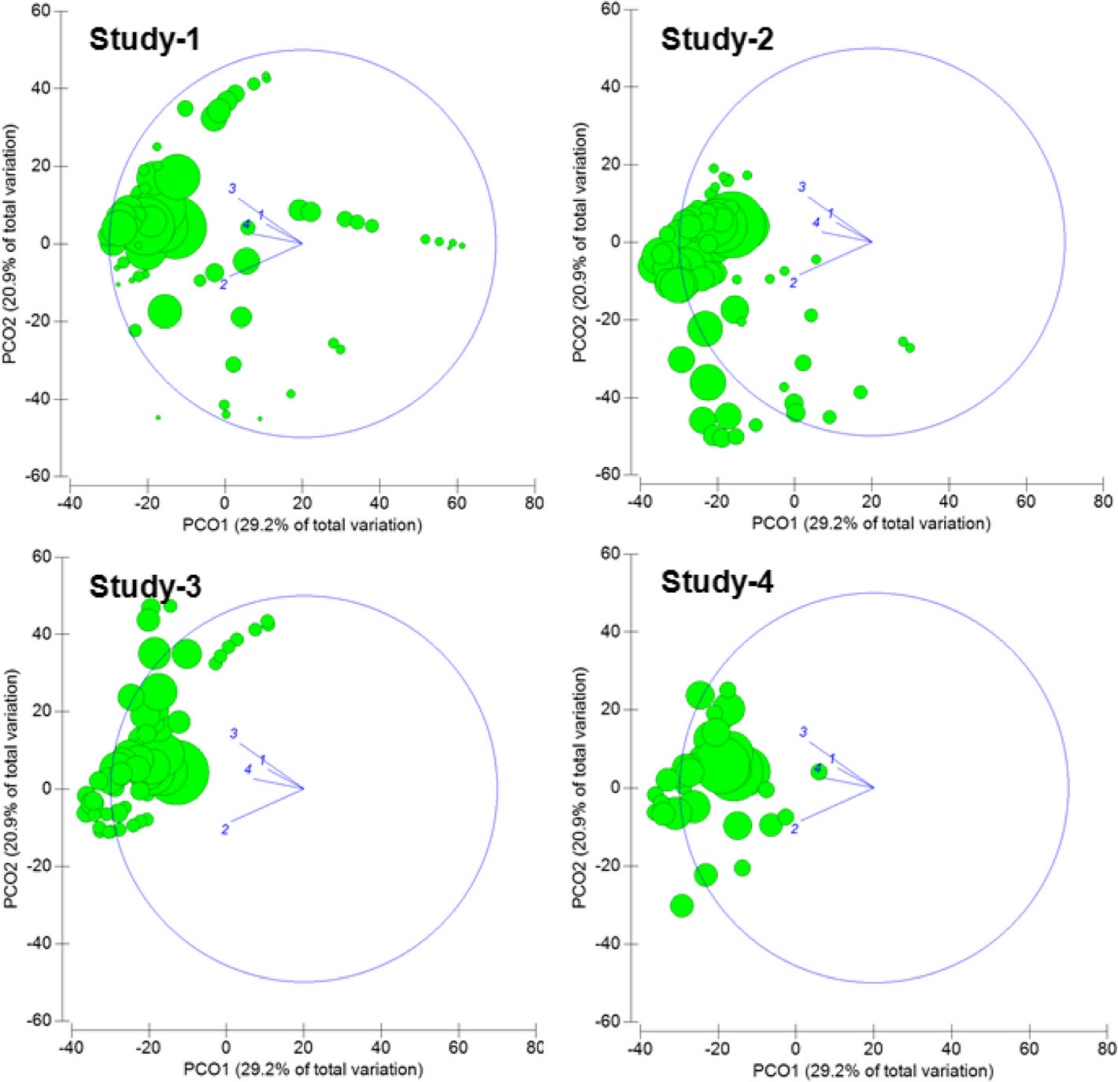
Principal coordinates analysis (PCoA) visualising the similarity of PCR-ribotype (PRT) distributions between the 4 studies. Each circle indicates a PRT; the size of the circle is proportional to the number of isolates. Plots show the PRT data cloud for the 4 studies. Each figure was derived from the same PCoA and thus positional differences between the data clouds indicate differences in PRT distribution. Bubbles occurring in the same location across PCO1 (X-axis) and PCO2 (Y-axis) indicate PRTs that were common to the different studies. The vector plot shows directional effects of the PRT distribution from each study

NTHi isolates were cultured from nasopharyngeal swabs or aspirates as described previously [4, 12]. Specimens were stored in skim milk-tryptone-glucose-glycerol broth at −70 to −80 °C prior to culture of 10 μl of specimen onto bacitracin-vancomycin-clindamycin chocolate agar. NTHi were identified by colony morphology, dependence on X and V factors, and a lack of reaction with the Phadebact® Haemophilus Test capsular antisera (MKL Diagnostics, Sweden). All isolates (Studies 3–4) or the majority of isolates representing all PRTs (Studies 1–2) were tested for *H. haemolyticus* misidentification by PCR targeting *hpd* or 16S rRNA genes [14, 15]. In the Northern Territory studies, 0.2 % (1/479) of presumptive NTHi from nasopharyngeal swabs were *H. haemolyticus* [16], while 9.5 % (57/595) of presumptive NTHi from Studies 3 and 4 in Western Australia were *H. haemolyticus* [14]. Isolates were not tested for Protein D expression or as capsule-deficient strains of encapsulated *H. influenzae.* For Study 1 and Study 2, one NTHi colony from each positive specimen, and any that were morphologically distinct, were genotyped by PRT [13]. For Study 3 and Study 4, two colonies of presumptive NTHi where available (including any that were morphologically distinct) were subjected to PRT. A single isolate of each PRT from each swab was included in the analysis. Principal coordinates analysis of genotyping data was performed using Plymouth Routines in Multivariate Ecological Research (PRIMER)-E (PRIMER-E Ltd, UK) [17]. The similarity of PRT distributions across the four studies is shown in Fig. 1, with circles indicating each PRT and its positional location across the principal coordinates (PCO1 and PCO2) indicated on the axes; the size of the circle is proportional to the number of isolates.

## Results and discussion

In the Northern Territory paediatric cohorts (Study 1 and Study 2), the mean NTHi carriage rates across all swabs were approximately 50 % (unpublished) for Study 1 and 85 % (269/316) at baseline for Study 2 [11] (Table 1). In the Kalgoorlie studies, NTHi carriage rates among the Aboriginal (Study 3) and non-Aboriginal (Study 4) children were 36.3 % (183/504) and 9.4 % (98/1045), respectively [14]. In Study 1, 84 different PRTs were identified among 2179 NTHi isolates. In Study 2, 73 different PRTs were identified among 551 NTHi isolates. In Study 3, 65 different PRTs were detected among 231 NTHi isolates, and 37 PRTs were detected among 109 isolates in Study 4.

Although many different NTHi PRTs were present in these studies (Table 1), several NTHi PRTs dominated (Table 2), irrespective of study setting, ethnicity, or presence of AOM. In each study, the six dominant PRTs accounted for 44 % (960/2179) (Study 1), 35 % (193/551) (Study 2), 35 % (81/231) (Study 3), and 46 % (50/109) (Study 4) of all isolates. PRTs 3 and 8 were the most common, featuring in all four populations and being represented by 5–11 % of all isolates. PRTs 15, 14, 13, and 4 were the next most common PRTs, each featuring in three of the four populations (Table 2). MLST analysis (http://pubmlst.org/hinfluenzae/) of at least one representative isolate of the six dominant PRTs in Study 1 and 2 found that all sequence types were described from the United States and Europe, with the exception of isolates representing PRTs 27, 34, and one isolate of PRT 8.

**Table 2:**
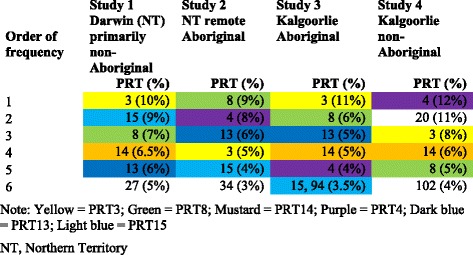
The 6 dominant non-typeable *Haemophilus influenzae* PCR-ribotypes (PRTs) in 4 populations of Australian children

Principal coordinates analysis (PCoA) was used to visualise the PRT distribution among the four study cohorts. This analysis showed a dominant cluster of PRTs common to all studies (Fig. 1), thereby supporting the absence of geographic or population-specific clustering of NTHi PRTs. The highest number of PRTs was detected in Study 1. Higher dispersion of PRTs across PCO1 and PCO2 for Studies 1–2 (compared to Studies 3–4) reflects the increased number of PRTs unique to Studies 1–2. For Study 1, this finding likely represents the large sample (2179 NTHi isolates). However, for Study 2, this finding likely reflects the larger number of strains dominating in this population, which may result from the high NTHi carriage rates (85 %) in this cohort or its broader geographic sampling (16 remote communities). This finding could also reflect a difference in disease state (all children had a diagnosis of AOM), though we previously showed that NTHi from paired nasopharyngeal and ear discharge swabs from children with AOM with perforation were generally the same PRT (unpublished observations).

**Fig. 2:**
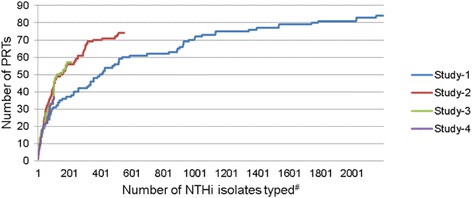
Rarefaction plot demonstrating the cumulative number of new non-typeable *Haemophilus influenzae* (NTHi) PRTs identified versus number of NTHi isolates typed for each study. PRT, PCR-ribotype. #Only unique isolates from each swab included

Analysis of these four diverse cohorts provided an opportunity to assess our sampling strategy for efficient surveillance of NTHi diversity within a population. Figure 2 shows the cumulative number of novel PRTs identified in each cohort against the number of NTHi isolates genotyped, irrespective of study duration. Studies 2, 3, and 4 followed a similar trajectory, in which 13–34 PRTs per 100 unique isolates were identified (Table 1). Study 2 was a 3-year study of 320 Aboriginal children up to 6 years of age, with NTHi carriage rates of up to 85 % at baseline; each child was swabbed up to three times over several weeks. Study 3 was a 6-year study of 100 Aboriginal children up to 2 years of age with a 36.3 % NTHi carriage rate; each child was swabbed up to 7 times over a 2-year period. Study 4 was the non-Aboriginal cohort of 180 children from Study 3 with a 9.4 % NTHi carriage rate. In contrast, Study 1 identified 4 PRTs per 100 isolates during a 6-month study of 456 non-Aboriginal children up to 4 years of age with an approximately 50 % NTHi carriage rate; each child was swabbed fortnightly. We have shown that low numbers of swabs with relatively low NTHi carriage (Study 3) collected less frequently and over a longer period can provide comparable information to intense, shorter studies such as Study 1.

Another carriage study design question relates to the number of isolates selected for analysis from each swab. Multiple NTHi genotypes during carriage have previously been observed and modelled [18]; however, the value of assessing multiple strain carriage in different populations is not well understood. In Studies 1–2 where additional colonies were selected only if morphologically distinct, multiple PRT carriage was detected in 8 and 17 % of swabs, respectively. For Studies 3–4 where 2 colonies were selected as per protocol, including any that were morphologically distinct, multiple PRT carriage was detected in 20 and 7 % of swabs, respectively. Although our data cannot inform the most appropriate number of colonies to select per specimen for measuring multiple PRT carriage, nor the method of selection, it is likely that these methods would vary by population. Indeed, the value of typing additional colonies appears to be greater for populations with high carriage rates.

In conclusion, analysis of NTHi nasopharyngeal carriage PRTs in four cohorts with varying NTHi colonisation rates allowed us to identify dominant genotypes common to four Australian paediatric populations. More than 3000 isolates from three distinct urban and remote geographic regions in Australia (Kalgoorlie in southern Western Australia, the urban Darwin region of the Northern Territory, and several remote communities across the Northern Territory) were examined. This finding provides an opportunity to study the factors conferring an advantage to the dominant PRTs in NTHi nasopharyngeal carriage, thereby advancing our understanding of the constraints that support NTHi population structure. Continued efforts to understand NTHi population dynamics will improve the identification of useful vaccine candidates for targeting universal or dominant strains, and may inform models of the potential efficacy of NTHi vaccines in current and future production pipelines. Further characterisation of carriage- and disease-related (acute [e.g. pneumonia] and chronic [e.g. bronchiectasis]) NTHi populations by whole genome sequencing or other genotyping means is necessary to determine the relevance of carriage versus disease genotype frequency, and may shed light on the factors supporting dominance of certain NTHi genotypes. Although the Australian experience may not necessarily apply elsewhere, whole genome sequence data from Australian and international NTHi isolates have (to date) demonstrated a level of core genome similarity between global and Australian NTHi populations [8]. Thus, we expect our findings to be applicable to other study settings.
